# An unusual tracheal foreign body in a middle-aged male with a 15-year history of coal use: a case report

**DOI:** 10.1186/s12880-021-00561-z

**Published:** 2021-02-23

**Authors:** Li-juan Zhong, Min Yan, Yi Wang, Dai-quan Zhou, Jian-ming Tang, Shou-hong Xiang

**Affiliations:** 1 Department of Radiology, The People’s Hospital of Leshan, Leshan City, 614000 People’s Republic of China; 2 Department of Radiology, The Third Affiliated Hospital of Chongqing Medical University (Gener Hospital), Chongqing, 401120 People’s Republic of China; 3 Department of Respiratory, The Third Affiliated Hospital of Chongqing Medical University (Gener Hospital), Chongqing, 401120 People’s Republic of China

**Keywords:** Tracheal foreign body, Coal dust inhalation, Computed tomography, Energy spectrum imaging

## Abstract

**Background:**

Long-term exposure to coal dust causes respiratory disease. In chest computer tomography (CT), pulmonary nodules, pulmonary interstitial fibrosis and emphysema manifest themselves. However, tracheal foreign bodies caused by coal dust are rarely reported. In this study, we report a special case of a tracheal coal foreign body, in which the patient has neither a history of coal work nor foreign body inhalation.

**Case presentation:**

A 49-year-old man was diagnosed with chronic obstructive pulmonary disease (COPD) due to chronic cough and exertional dyspnoea. His symptoms gradually worsened despite treatment for COPD. Chest radiograph and CT images showed an irregular high-density nodule inserting fromthe trachea into the right thyroid at approximately the level of the 7th cervical vertebra. Fiberoptic bronchoscopy revealed that the tracheal lumen was mostly blocked. After the surgery, the energy spectrum CT quantitative analysis showed that the foreign body was likely that of a bituminous coal specimen.

**Conclusions:**

For cases in which a foreign body in the airway is highly suspected, early fiberoptic bronchoscopy and radiographic examinations should be performed as soon as possible to avoid misdiagnosis and ensure timely treatment.

## Background

Tracheal foreign bodies are not uncommon, especially in kids and the elderly [1], however, a tracheal coal foreign body due to coal dust inhalation is very rare. To our knowledge, a tracheal coal foreign body has not previously been reported. The rarity of this foreign body and its unusual clinical manifestations make radiological and clinical diagnosis very difficult. We report here the first case of a tracheal coal foreign body, focusing on its appearance in radiological and clinical manifestations. The patient was a middle-aged man with a history of chronic cough and exertional dyspnea, but without fever. The foreign body was removed surgically and the patient's symptoms of dyspnea were therefore completely relieved. In a patient with a 15-year history of domestic coal use, a comprehensive analysis of fiberoptic bronchoscopy, surgical findings and energy spectrum computed tomography (CT) quantitative parameters established the diagnosis of a bronchial coal foreign body.

## Case presentation

A 49-year-old man with repeated cough and exertional dyspnea for 1 year was admitted to our hospital on January 8, 2019. Dating back to the patient’s clinical history at the local hospital, previous chest CT images showed a “tracheal foreign body”, but no specific foreign body was found by laryngoscope. Thus, the patient was only treated with chronic obstructive pulmonary disease (COPD) at that time. He had a 20-year history of one pack a day tobacco smoking. In fifteen out of the twenty years before hospital admission, the patient was exposed to coal dust without any respiratory protective equipment because coal was his primary source of winter heating (4 to 5 months per year). With an improvement in living conditions, he was not exposed to coal dust in the past five years. The patient denied having a history of coal work and large foreign body inhalation.

The patient’s wheezing could be heard in the suprasternal fossa when the patient inhaled and exhaled, but the three depressions sign was negative. The other examinations were unremarkable. Pulmonary function tests showed mild obstructive ventilatory dysfunction and a decrease in small airway function. The values of FEV_1_/FVC and FEV_1_% predicted were 68.64% and 76.6%, respectively. The bronchial dilation test was negative.

A routine blood test showed that white blood cells were 9.39 × 10^9^/L, with the differential of 88.8% neutrophils and 7.0% lymphocytes. There were no other significant abnormalities in residual blood and biochemical indicators. The tumor markers and sputum samples for tuberculosis were all negative.

**Fig. 1:**
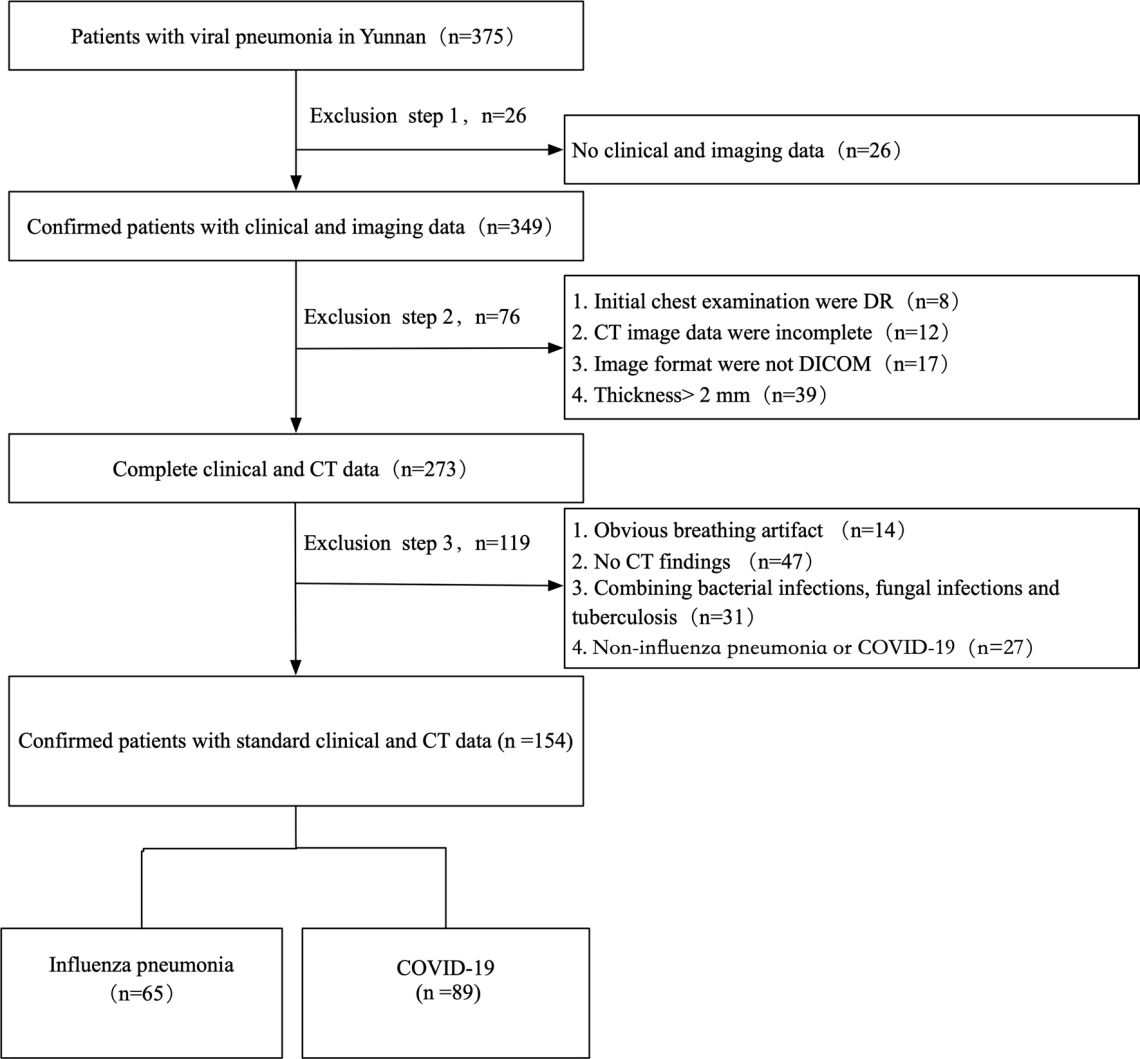
Radiological examinations and fiberoptic bronchoscopy were performed on a 49-year-old man with a tracheal coal foreign body prior to surgery. **a** Chest radiograph shows a high-density nodule (arrows) in the trachea about the level of the 7th cervical vertebra. **b**, **c** Chest axial and coronal CT images also reveal this high-density nodule (arrows) inserted from the trachea into the right thyroid at the same level as seen on the chest radiograph. **d** Fiberoptic bronchoscopy reveals a black foreign body in the subglottic trachea. The tracheal lumen was mostly blocked and the fiberoptic bronchoscopy failed to pass through the trachea due to its severe stenosis

The admission chest radiograph showed a high-density nodule in the trachea at approximately the level of the 7th cervical vertebra, while there was no obvious abnormality found in either lung (Fig. 1a). Chest CT images also revealed a high-density nodule in the trachea at the same level as seen on the chest radiograph. The image showed the nodule inserting into the right thyroid. The foreign body was well-defined, irregular, about 35 mm × 19 mm × 25 mm in size, and measured 1750 HU for its CT attenuation value. The widest space between the tracheal wall and the foreign body was only about 6.5 mm (Fig. 1b, c). Fiberoptic bronchoscopy revealed a black foreign body with an irregular shape in the subglottic trachea. The tracheal lumen was mostly blocked, and granulomatous hyperplasia could be seen at the interface between the foreign body and the tracheal mucosa. However, the fiberoptic bronchoscope failed to pass through the trachea due to its severe stenosis (Fig. 1d).

The patient was placed in the supine position and general anesthesia with extracorporeal membrane oxygenation (ECMO) support was administered. A transverse incision was made in the neck about 3 cm above the superior sternal fossa to separate the banded muscle and the tissues around the anterior wall of the trachea. Following a cut from the 4th to 5th tracheal ring and the creation of a U-shaped fistula to insert the endotracheal catheter, the ventilator was connected. Through the incision, the bilateral thyroids were exposed. A black tracheal foreign body penetrated the right wall of the trachea and inserted into the lower pole of the right thyroid. The cicatrization in the thyroid tissue around the foreign body was very serious, and required the resection of most of the right thyroid. A cut from the 1st to 2nd tracheal ring exposed the black foreign body. The irregularly-shaped foreign body was fixed and could not be removed as a whole. A clamp was used to clip the foreign body into pieces and remove them one by one until there was no foreign body residue remaining. We explored the trachea again and observed a proliferation of granulation tissue around the rupture in the right tracheal wall. The tracheal rupture was sutured with Prolene sutures and covered with a neck muscle flap. The neck was then sutured by the standard fashion. The patient recovered uneventfully and his dyspnea was completely relieved after operation.

**Fig. 2:**
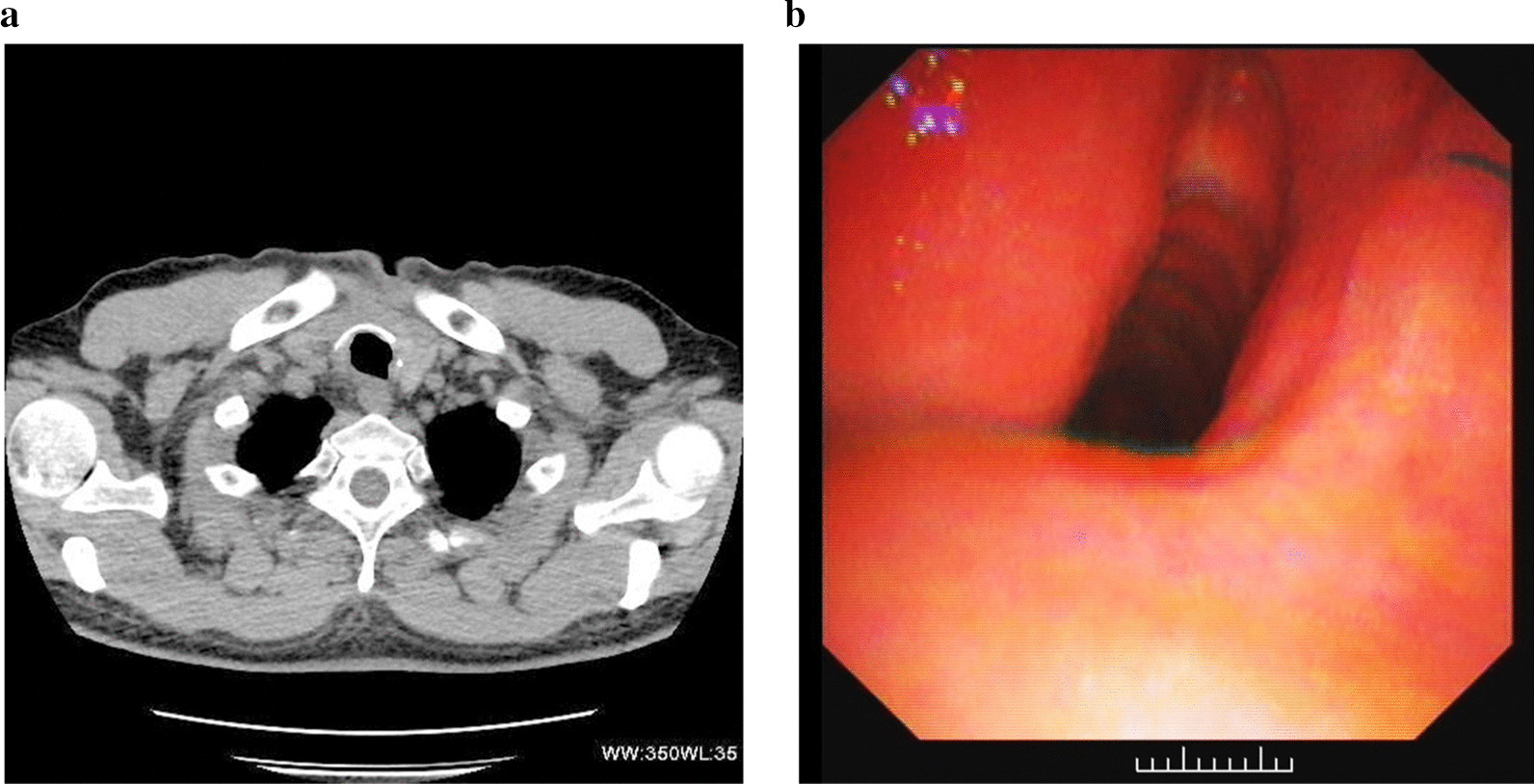
The patient again underwent CT examination and fiberoptic bronchoscopy one month after the operation. **a** An axial CT image shows that the tracheal cavity was regular after the original foreign body was removed. **b** Fiberoptic bronchoscopy reveals that the tracheal cavity was slightly narrow, but the tracheal mucosa was normal

In follow-up treatment one month after the operation, the patient underwent a chest CT and fiberoptic bronchoscopy. No tracheal abnormality was found on the CT images and the fiberoptic bronchoscopy only showed a slight tracheal stenosis at the original surgical site (Fig. 2a, b). In the subsequent 3-month follow-up period, the patient reported no acute clinical episodes, and no general problems in his work or ordinary activities.

**Fig. 3:**
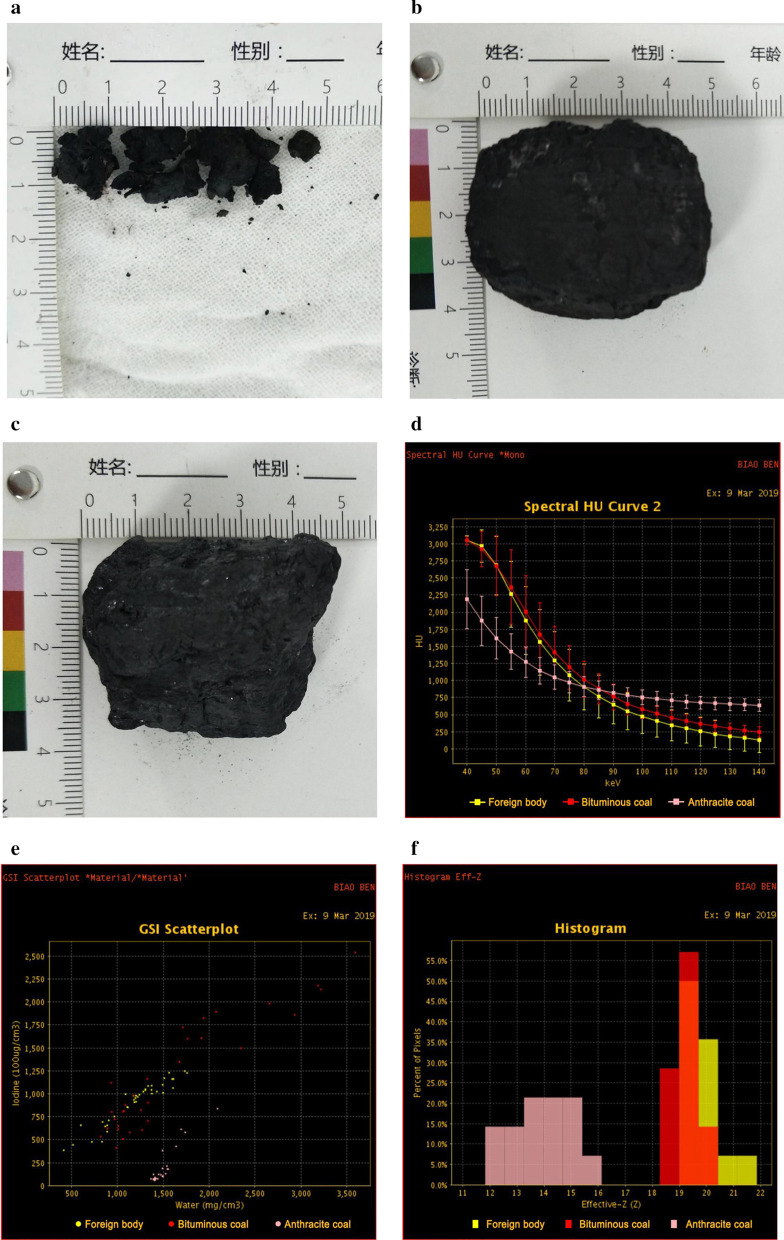
Energy spectrum CT was performed to quantitatively measure and compare spectral parameters of the surgically extracted foreign body with bituminous and anthracite coals. **a** Specimens from the foreign body. **b** Bituminous coal sample. **c** Anthracite coal sample. **d–f** Energy spectrum CT images show that there was very good agreement of the spectral HU curves (**d**), GSI scatterplot (**e**) and effective Z-value (**f**) between the foreign body and the bituminous coal, but poor agreement from those of the anthracite coal

To further identify properties of the tracheal foreign body (Fig. 3a), we compared it with samples of bituminous (Fig. 3b) and anthracite (Fig. 3c) coals and performed a quantitative analysis of energy spectrum CT (Revolution CT, GE Healthcare, Milwaukee, Wisconsin). Spectral HU curves and the gemstone spectral imaging (GSI) scatterplot showed very good agreement between the foreign body and the bituminous coal, but low agreement between the sample and the anthracite coal (Fig. 3d, e). The results of effective Z-value analysis showed that the distribution range of the foreign body was 19.0–21.8 and the highest peak was 19.0–19.6, which were also highly similar to the distribution range (18.4–20.4) and the highest peak (19.0–19.6) of the bituminous coal, but different from the distribution range (11.8–16.2) and the highest peak (13.2–15.4) of the anthracite coal (Fig. 3f).

## Discussion and conclusion

Coal workers with chronic exposure to coal dust often present a variety of respiratory illnesses. The main disease is pneumoconiosis and the most common complication is pulmonary tuberculosis [2]. Therefore, in previous reports [3], chest radiographs and CT images often showed multiple pulmonary nodules, interstitial fibrosis and emphysema. In the current case, it is very unusual that, although the patient had been exposed to coal dust for fifteen years, chest X-ray and CT examinations only revealed an irregular high-density nodule in the trachea, and there was no obvious abnormality found in either lung. To our knowledge, such a rare situation has not previously been reported.

Based on imaging and the patient's medical history, we speculated that the foreign body may be coal. Fiberoptic bronchoscopy and surgery revealed that the foreign body was a black substance, which supported our initial speculation. After surgery, we used energy spectrum CT to quantitatively measure and compare the spectral parameters of the specimens from the foreign body with bituminous and anthracite coal samples. The results showed that there was a very good agreement of the spectral HU curves, GSI scatterplots and effective Z-values between the foreign body and the bituminous coal. Upon further consultation with the patient, he recalled using bituminous coal as a heating source every winter for fifteen years. Therefore, we determined that the foreign body was likely the deposition of bituminous coal dust.

Regarding the formation of the foreign body, we have made the following speculations. First, after inhalation, coal dust may have accumulated on the tracheal mucosa or sputum bolt in such a way it could not attach to the tracheal wall via coughing. The foreign body gradually increased in size as the exposure time increased. Second, there may have been an existing ulcer or injury on the patient’s tracheal wall, which aggravated the inflammatory response of the coal dust inhalation, leading to local fibrosis and additional deposition of coal dust. Third, the patient’s tracheal wall may have been unknowingly pierced with an object such as bone, wood or coal filings that lodged itself in the trachea and acted as a support point for coal dust accumulation. Several previous studies have reported that various foreign bodies, including plastic discs and bones, have entered the trachea without the patients having any recollection of inhaling them [4–6]. As a result of the inhaled coal dust and gradual accumulation of tracheal secretions, an irregular coal foreign body formed. The patient’s daily activities and often severe cough may have caused the foreign body to penetrate the tracheal wall and insert into the right thyroid.

A clinical finding of roughly 85% tracheal obstruction with no obvious accompanying acute respiratory symptoms is rare. Generally, severe obstruction of the airway by a tracheal foreign body may cause hypoxemia-induced rapid cardiac arrest [7]. However, in the current case, the patient reported only repeated coughing and exertional dyspnea. The patient developed exertional dyspnea only one year prior to our intervention. It may be that, during the formation of the foreign body, the tracheal lumen was gradually narrowed at a slow enough rate that the body adapted accordingly to the increasing hypoxic state. Below a certain threshold of space between the foreign body and the tracheal wall, oxygen intake was not sufficient, and the symptoms of exertional dyspnea occurred. Macchiarini [8] suggested that, when the tracheal lumen narrows to less than 8 mm in diameter, exertional dyspnea will not develop; however, when the lumen is smaller than 5 mm, dyspnea will occur at rest. In the current case, the narrowest tracheal lumen diameter was approximately 6.5 mm and the symptoms of the patient were manifested as exertional dyspnea and occasional nocturnal dyspnea, largely agreeing with Macchiarini’s findings.

For the diagnosis of the tracheal foreign body, radiological examinations and bronchoscopy are necessary. With the development of CT post-processing techniques, some authors have reported a detection rate of 100% for tracheal foreign bodies with CT with multiplanar reformation (MPR) [9]. CT with MPR can clearly indicate the anatomical relationship between the foreign body, the tracheal wall and its adjacent tissues, and accurately evaluate the severity of tracheal stenosis [10]. The golden standard for the diagnosis of airway foreign bodies is bronchoscopy, which can be not only a diagnostic tool but also an effective aid in removing tracheal foreign bodies [11]. Because the foreign body was relatively large, fixed, and inserting into the right thyroid, the fiberoptic bronchoscope failed to remove it. Fortunately, subsequent surgery successfully removed the foreign body, and the patient's symptoms of dyspnea were completely relieved.

In conclusion, we have reported on a very unusual case of a tracheal foreign body caused by coal dust inhalation in a middle-aged male patient. To our knowledge, this is the first case reported in the literature in which a tracheal coal foreign body was discovered in chest radiographs and CT images, and energy spectrum CT was used in the identification of the foreign body. In the process of diagnosis, chest CT provided accurate and reliable clinical diagnostic information and played an important role in determining how to remove the tracheal foreign body. Quantitative analysis of the energy spectrum CT and the detailed medical history provided important evidence for identifying the properties of the foreign body. For patients in whom an airway foreign body is suspected, fiberoptic bronchoscopy should be performed as soon as possible to ensure proper treatment.

## Data Availability

The datasets used during the current study are available from the corresponding author on reasonable request.

## Abbreviations

CT: Computed tomography
COPD: Chronic obstructive pulmonary disease
FEV_1_: Forced expiratory volume in 1 s
FVC: Forced vital capacity
ECMO: Extracorporeal membrane oxygenation
GSI: Gemstone spectral imaging
MPR: Multiplanar reformation

## References

[1] Sun YL, Bao Z, Wang XF, Wang LH, Zhou JY (2016). The tracheobronchial foreign body in welder without the history of allotriophagy and foreign body aspiration. Clin Respir J.

[2] Jin Y, Wang H, Zhang J, Ding C, Wen K, Fan J, Li T (2018). Prevalence of latent tuberculosis infection among coal workers' pneumoconiosis patients in China: a cross-sectional study. BMC Public Health.

[3] Laney AS (2014). Respiratory diseases caused by coal mine dust. J Occup Environ Med.

[4] Ciolek PJ, Lorenz RR (2017). Misdiagnosis of a tracheal foreign body of decades-long duration. JAMA Otolaryngol Head Neck Surg.

[5] Gonzalez L, Candelario A, Otero Y, Torres-Luna L, Cantres O, Rodriguez-Cintron W (2019). Chronic nonasphyxiating bronchial foreign body removal: bronchoscopic debridement with tracheal window extraction. J Bronchology Interv Pulmonol.

[6] Gray G, Adams M, Black M, Sidhu P (2019). Removal of an inhaled stoma button distal to a reactionary tracheal stenosis: a difficult airway case. BMJ Case Rep.

[7] Mohammad M, Saleem M, Mahseeri M, Alabdallat I, Alomari A, Za'atreh A, Qudaisat I, Shudifat A, Nasri AM (2017). Foreign body aspiration in children: a study of children who lived or died following aspiration. Int J Pediatr Otorhinolaryngol.

[8] Macchiarini P (2006). Primary tracheal tumours. Lancet Oncol.

[9] Yang Y, Liu YH, Cheng Q, Cheng Z, Wu SH, Ding D, Xu SC (2017). Application of MDCT and post-processing in children with tracheal foreign body. Lin Chung Er Bi Yan Hou Tou Jing Wai Ke Za Zhi.

[10] Ferretti GR, Kocier M, Calaque O, Arbib F, Righini C, Coulomb M, Pison C (2003). Follow-up after stent insertion in the tracheobronchial tree: role of helical computed tomography in comparison with fiberoptic bronchoscopy. Eur Radiol.

[11] Xiang Z, Ai Z, Zhong G, Deng Y, Malhi H, Palmer S, Zee C (2017). Diagnostic value of using multiplanar reformation images: case report for rare endotracheal hamartomas. Medicine (Baltimore).

