# Correction to: An unusual tracheal foreign body in a middle-aged male with a 15-year history of coal use: a case report

**DOI:** 10.1186/s12880-021-00591-7

**Published:** 2021-04-06

**Authors:** Li-juan Zhong, Min Yan, Yi Wang, Dai-quan Zhou, Jian-ming Tang, Shou-hong Xiang

**Affiliations:** 1 Department of Radiology, The People’s Hospital of Leshan, Leshan City, 614000 People’s Republic of China; 2 Department of Radiology, The Third Affiliated Hospital of Chongqing Medical University (Gener Hospital), 401120, Chongqing, People’s Republic of China; 3 Department of Respiratory, The Third Affiliated Hospital of Chongqing Medical University (Gener Hospital), 401120, Chongqing, People’s Republic of China

## Correction to: BMC Med Imaging (2021) 21:35 10.1186/s12880-021-00561-z

Following the publication of the original article [1] the authors informed us that an incorrect image had unfortunately been included as Fig. 1 in their article.

The correct Fig. 1 is shown here below and has now been updated in the original article.

**Fig. 1:**
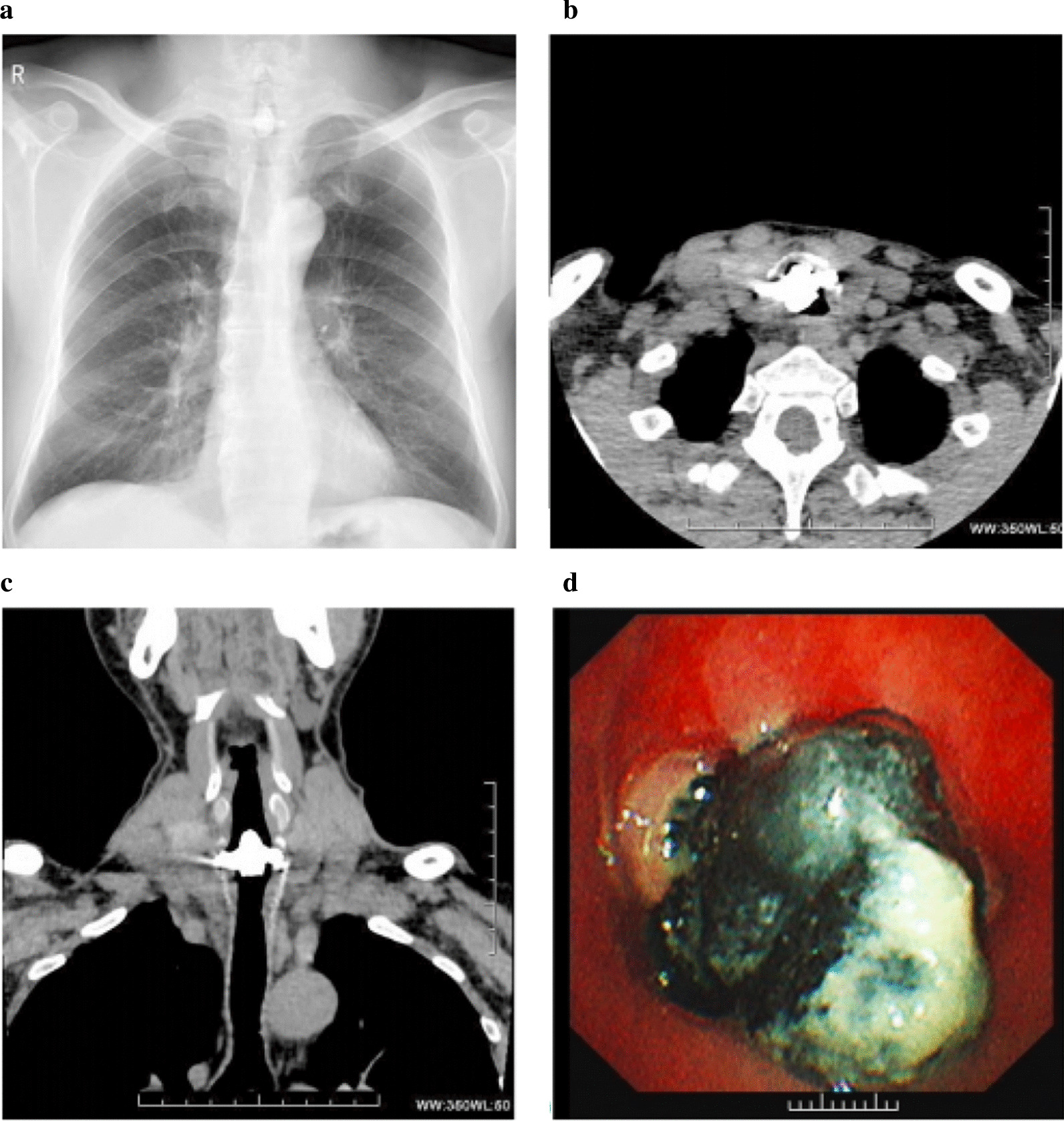
Radiological examinations and fiberoptic bronchoscopy were performed on a 49‑year‑old man with a tracheal coal foreign body prior to surgery. **a** Chest radiograph shows a high‑density nodule (arrows) in the trachea about the level of the 7th cervical vertebra. **b**, **c** Chest axial and coronal CT images also reveal this high‑density nodule (arrows) inserted from the trachea into the right thyroid at the same level as seen on the chest radiograph. **d** Fiberoptic bronchoscopy reveals a black foreign body in the subglottic trachea. The tracheal lumen was mostly blocked and the fiberoptic bronchoscopy failed to pass through the trachea due to its severe stenosis
